# Harmine Plus Exendin-4 Enhances Remission of Recent-Onset Type 1 Diabetes Following Anti-CD3 Therapy

**DOI:** 10.64898/2026.09.21.753317

**Published:** 2026-09-22

**Authors:** Geming Lu, Randy Kang, Miguel Varela, Eunjin Oh, Yansui Li, Jungeun Lee, Leah Kebrom, Juan Aldaco, Jiamin Zhang, Madeline Wichman, Mingpei Li, Fouad Kandeel, Meirigeng Qi, Peng Wang, Robert J. DeVita, Alberto Pugliese, Debbie C. Thurmond, Andrew F. Stewart, Adolfo Garcia-Ocana

## Abstract

Type 1 diabetes (T1D) results from autoimmune destruction of pancreatic β-cells. While anti-CD3 therapy can delay disease progression and preserve residual β-cell function, disease reversal will likely require both immune modulation and β-cell regeneration. We found that the combination of harmine and exendin-4 (H+E) reduced inflammation-induced human β-cell apoptosis, suppressed cytokine signaling and immunogenicity pathways, and improved β-cell function. Although H+E alone did not reverse diabetes in NOD mice, low-dose anti-CD3 followed by H+E normalized blood glucose, increased insulin levels, improved glucose tolerance, expanded β-cell mass, and enhanced diabetes remission. These effects were associated with reduced pro-inflammatory T-cell responses, increased regulatory T cells, and greater expression of exhaustion-related T-cell markers, without broad lymphocyte depletion. Similar immunomodulatory effects were observed in activated human PBMCs. Transcriptomic analyses identified the lncRNA SNHG6 as a key mediator of H+E action; SNHG6 protected β-cells from cytokine-induced stress, apoptosis, and immunogenicity. Together, these findings demonstrate that H+E promotes β-cell recovery and resilience while reducing β-cell immunogenicity, enabling remission of recent-onset T1D when combined with anti-CD3 therapy. SNHG6 emerges as a novel regulator of β-cell protection during inflammation.

## Full Text Availability

The license terms selected by the author(s) for this preprint version do not permit archiving in PMC. The full text is available from the preprint server.

